# Immunoendocrine Peripheral Effects Induced by Atypical Antipsychotics

**DOI:** 10.3389/fendo.2020.00195

**Published:** 2020-04-21

**Authors:** Samantha Alvarez-Herrera, Raúl Escamilla, Oscar Medina-Contreras, Ricardo Saracco, Yvonne Flores, Gabriela Hurtado-Alvarado, José Luis Maldonado-García, Enrique Becerril-Villanueva, Gilberto Pérez-Sánchez, Lenin Pavón

**Affiliations:** ^1^Laboratorio de Psicoinmunología, Dirección de Investigaciones en Neurociencias del Instituto Nacional de Psiquiatría Ramón de la Fuente Muñiz, Ciudad de México, Mexico; ^2^Clínica de Esquizofrenia, Instituto Nacional de Psiquiatría Ramón de la Fuente Muñiz, Ciudad de México, Mexico; ^3^Laboratorio de Investigación en Inmunología y Proteómica, Hospital Infantil de México Federico Gómez, Ciudad de México, Mexico; ^4^Area of Neurosciences, Department of Biology of Reproduction, CBS, Universidad Autonoma Metropolitana-Iztapalapa, Mexico City, Mexico

**Keywords:** atypical antipsychotics (AAP), peripheral effects, inflammatory response, endocrine response, microbiome

## Abstract

Atypical antipsychotics (AAP) or second-generation antipsychotics are the clinical option for schizophrenia treatment during acute psychoses, but they are also indicated for maintenance during lifetime, even though they are being used for other psychiatric conditions in clinical practice such as affective disorders and autism spectrum disorder, among others. These drugs are differentiated from typical antipsychotics based on their clinical profile and are a better choice because they cause fewer side effects regarding extrapyramidal symptoms (EPS). Even though they provide clear therapeutic benefits, AAP induce peripheral effects that trigger phenotypic, functional, and systemic changes outside the Central Nervous System (CNS). Metabolic disease is frequently associated with AAP and significantly impacts the patient's quality of life. However, other peripheral changes of clinical relevance are present during AAP treatment, such as alterations in the immune and endocrine systems as well as the intestinal microbiome. These less studied alterations also have a significant impact in the patient's health status. This manuscript aims to revise the peripheral immunological, endocrine, and intestinal microbiome changes induced by AAP consumption recommended in the clinical guidelines for schizophrenia and other psychiatric disorders.

## Introduction

Antipsychotics have been widely used in clinical psychiatry and neuroscience research for over 68 years since chlorpromazine demonstrated sedative effects in psychotic patients (1). Antipsychotics drugs are classified as typical or atypical according to the clinical effects that they cause (2). Atypical (AAP) or second-generation antipsychotics (SGA) are effective against positive and negative symptoms and improve some domains of cognition of schizophrenia. AAP are the first clinical option to treat various psychiatric conditions because they produce significantly fewer EPS and pose a lower risk of pseudo-parkinsonism and catalepsy in comparison to typical antipsychotics (3, 4).

Even though AAP were initially prescribed for psychotic disorders like schizophrenia, the Food and Drug Administration (FDA) has approved the use of these drugs for the treatment of other psychiatric conditions, including bipolar disorder, major depressive disorder with psychotic features, acute agitation, Tourette syndrome, borderline personality disorder, dementia, and substance-induced psychotic disorder (5) as well as diagnosed psychiatric conditions in children (6).

The pharmacological and adverse effects related to AAP consumption are due to the affinity of these drugs to a broad range of neurotransmitter receptors located in the CNS, peripheral organs, tissues, and cells. Each AAP has its own unique affinity pattern that generates psychiatric and peripheral effects acting at dopamine (DA) D1, D2, D3, D4, adrenergic α-1 and α-2, serotoninergic 5-HT_2A_ and 5-HT_2C_, histaminergic, and muscarinic receptors (7). Despite their enormous efficacy on psychiatric symptoms and their low rate of EPS, AAP are not without adverse side effects. It is well-known that AAP produce peripheral effects related with metabolic alterations (8) like weight gain, type 2 diabetes, dyslipidemia, and subsequent cardiovascular complications (9, 10).

However, AAP consumption induces other peripheral changes that are clinically relevant but commonly dismissed, such as alterations in the immune and endocrine function as well as the intestinal microbiome. These sets of changes play a significant role in triggering inflammatory and metabolic chronic changes that affect the adequate recovery of patients and their quality of life.

A wide variety of hormones show alterations in their circulatory levels in human and animal models during AAP consumption. Among the affected hormones are those related to glucose metabolism, orexigenic and anorexigenic molecules, and hormones secreted by the hypothalamus or pituitary (11–14).

In patients and experimental models, AAP consumption modifies leukocyte phenotype, and cell count. The evidence demonstrates that macrophages (MQs), dendritic cells (DCs), T and B lymphocytes, neutrophils, and other leukocytes modify their function as well as cytokine production and release, apoptosis, phagocytosis, and Th1-Th2 differentiation (15–17). Additionally, other reports show AAP can change the peripheral levels of pro-inflammatory, anti-inflammatory, and growth factors molecules like C-reactive protein (CRP), interleukin (IL)-1β, IL-6, IL-12, IL-10, tumor necrosis factor (TNF)-α, interferon (IFN)-γ, and other molecules, affecting the systemic condition of the organism (18, 19).

Changes in hormonal and inflammatory levels in patients that consume AAP impact intestinal microbiota. It is important to note that the growing evidence suggests the intestinal microbiome could be involved in the treatment response. Moreover, gut microorganisms might be necessary to the occurrence of adverse effects such as weight gain (20).

In this review, we summarize the clinical and experimental studies that demonstrated the immunological, endocrine, and intestinal microbiome changes induced by the consumption of each AAP approved by the FDA for the treatment of schizophrenia and other psychiatric disorders.

### Why Is It Important to Make Evident the Neuroendocrine Effects Induced by AAP Consumption?

Antipsychotic therapy prevails as a standard and fundamental component for major psychiatric disorders like acute episode of psychoses and the maintenance phase of schizophrenia and schizoaffective disorders (21). These drugs do not act exclusively on the CNS, and the more evident problem related with AAP treatment is the higher risk of developing hyperphagia, hyperglycemia, dyslipidemia, weight gain, diabetes mellitus, and insulin resistance (22), which further develops metabolic and cardiac complications with subsequent reduction in life expectancy, poor patient compliance, and sudden death (4).

Daily clinical practice not only identifies metabolic problems but also three clinically relevant issues that are the result of the chronic consumption of AAP by patients (23), often disregarded by healthcare professionals. The first are the endocrine and immune effects that AAP cause in patients and that will be listed in subsequent sections. The second is that the effects of the cotreatment with AAP and psychiatric medication, despite being more common than which is acknowledged (approximately 66% of psychiatrists use AAPs in combination), (24). The most common causes of co-treatment are: Patients seemed resistant to treatment instead of a monotherapy assay with clozapine or had been diagnosed with two or more psychiatric diagnoses, the clinician overlapped one antipsychotic while another was titrated (switching medication because of lack of response or better security profile), and finally, an effective dose of an AP was not achieved because of intolerance or side effects. Finally, the third issue is the joint effect of AAP consumption together with other drugs as benzodiazepines (adjunctive therapy for acute agitation, comorbid anxiety, or distress), antidepressants (as the adjunctive therapy in schizophrenia for persistent negative symptoms, comorbid major depressive disorder, and suicide risk) (25), APs also are used as adjunctive therapy for treatment-resistant major depressive disorder and major depressive disorder with psychotic features, and mood stabilizers as adjunctive therapy in bipolar disorder, schizoaffective disorder, and ultra-resistant schizophrenia (26).

This evidences the wide therapeutic use of AAP, which have become first-choice drugs to treat schizophrenia and other psychoses due to the lower risk of developing EPS. However, these drugs are highly promiscuous in their interaction with several neurotransmitter receptors as 5-HT and D, expressed in different peripheral cell types, such as leukocytes and gland cells (27–30), which constitutively express these receptors AAP can bind to.

**Figure 1:**
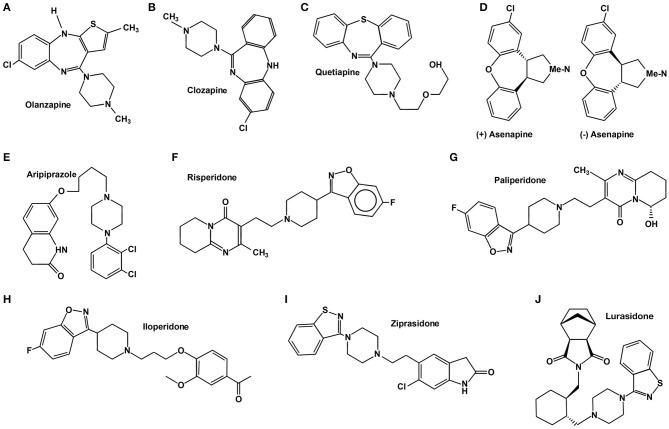
Chemical structure of atypical antipsychotics.

To understand the diversity of effects these drugs systemically induce, we must consider that AAP do not behave equivalently, as shown in Figure 1. They are structurally heterogeneous, and the therapeutic effects, albeit generally equivalent, have particularities firstly explained by their heterogeneous physicochemical interactions with several receptors (see Table 1). The interactions between these drugs and their receptor firstly induces the conformational changes within the receptor structure that result in the activation of the associated heterotrimeric G protein (GPCR) and its consequent activation (53).

**Table 1:** Characteristics of APPs interaction with different neurotransmitter receptors.

	**M1**	**1/2**	**D1**	**1/2**	**D2**	**1/2**	**D3**	**1/2**	**D4**	**1/2**	**5HT_**1A**_**	**1/2**	**5HT_**2A**_**	**1/2**	**5HT_**2C**_**	**1/2**	**5HT_**6**_**	**1/2**	**5HT_**7**_**	**1/2**	**α 1A**	**1/2**	**α2A**	**1/2**	**H1**	**1/2**	**References**
Olanzapine	73.0	1	11.0	2	14.4	2	43.0	2	50.0	2	3442.0	1	4.0	2	11.00	2	5.00	2	nd	-	19.0	2	nd	-	7.0	2	(31–36)
Clozapine	6.1	2	266.2	1	157.0	1	269.0	1	26.3	1	123.7	2	5.3	1	9.44	1	13.4	1	17.9	1	1.6	1	37.0	2	1.1	1	(36, 37)
Quetiapine	858.0	1	712.0	1	245.0	1	483.0	1	1202.0	1	432.0	2	101.0	1	2502.0	1	1865.0	1	307.0	1	22.0	1	3630.0	1	11.0	1	(36, 38)
Asenapine	-	-	1.4	1	1.3	1	0.4	1	1.1	1	2.5	1	0.1	1	0.03	1	1.10	1	1.4	1	1.2	1	1.2	1	1.0	1	(39)
Aripiprazole	6780.0	nd	265.0	nd	66.0	2	0.8	2	44.0	2	5.5	2	8.7	1	22.00	2	214.0	1	9.6	1	26.0	1	74.0	1	30.0	nd	(40–42)
Risperidone	>10,000	-	580.0	1	3.2	1	18.0	1	22.0	1	282.0	1	0.5	2	19.00	2	4118.0	1	3.5	1	8.0	1	9.5	1	34.0	2	(36, 43)
Paliperidone	>10,000	-	554.0	1	2.8	1	7.5	1	38.0	1	1030.0	1	0.8	2	19.00	2	3425.0	1	3.8	1	11.0	1	111.0	1	34.0	2	(44, 45)
Iloperidone	nd	-	216.0	2	7.1	2	7.1	2	25.0	2	168.0	1	5.6	2	14.00	2	43.0	2	22.0	2	>10000	2	162.0	2	437.0	2	(46–51)
Ziprasidone	300.0	nd	130.0	nd	4.8	1	7.2	1	105.0	1	76.0	2	1.4	1	13.00	2	76.0	1	9.3	1	18.0	1	160.0	1	130.0	1	(52)
Lurasidone	>1,000	nd	262.0	nd	1.6	1	15.7	1	30.0	nd	6.7	2	2.0	1	415.00	nd	-	-	0.5	1	48.0	nd	10.8	nd	>1000	nd	(32)

*The binding affinity (Ki) are expressed in nM by each AAPs and receptor when the information was available. 1 = antagonist; 2 = agonist; nd = non determined*.

In addition, over the past decade new mechanisms associated with GPCR function have been discovered, such as the ability of β-arrestins to act as multifunctional proteins and activate multiple mediators like ERK, proto-oncogene tyrosine-protein kinase SRC, nuclear factor-κB, and phosphoinositide 3-kinase (54). The capacity of a ligand to preferentially activate either G protein-dependent signaling or G protein-independent signaling is called “biased agonism” or “functional selectivity.” This innovative new concept reflects the heterogeneity and complexity of the different receptor conformation states it can be transitioning when specifically interacting with stimulants (55). In addition, recent data have demonstrated how receptor functional selectivity is a dynamic and adaptable process, which can also be modified by physiopathological conditions (56).

In addition, it must be considered that, in cases such as cotreatment, the combined effect of two or more AAP or polypharmacy can induce a phenomenon called Convergence of signaling pathways occurring in cells. It can change the overall outcome of signals initiated with different relative strengths of signal, while initial events at the cell membrane may also pose differential consequences for the whole cell (57). These phenomena are reflected in the fluctuations of soluble mediators such as cytokines and hormones in AAP consumers.

### Immunoendocrine Peripheral Effects Induced by AAP

In this section we will exhibit the specific immunoendocrine peripheral effects of the 10 most prescribed APs drugs approved by the FDA: olanzapine, clozapine, quetiapine, asenapine, aripiprazole, risperidone, paliperidone, iloperidone, ziprasidone, and lurasidone.

## Clozapine

Clozapine was the first AAP developed in 1958 and it was approved by the FDA in 1989 after 31 years of investigations and clinical trials for treatment-resistant schizophrenia (58). Currently, it is considered to be one of the most effective antipsychotics for the treatment of schizophrenia, psychosis, and depression. Nevertheless, it is not the first-line drug of choice due to its range of adverse effects, making compliance an issue for many patients (59).

Clozapine is often discontinued (18) since it has some other potentially dangerous and life-threatening side effects, such as myocarditis, seizures, agranulocytosis, or granulocytopenia, and gastrointestinal hypomotility. It is a 5-HT_2A_ and D4 receptor antagonist. It also shows affinity to D1, D2, D3, D5, α-adrenergic, histaminergic H1, and cholinergic receptors (37) (see Table 1). This fact hinders the understanding of its molecular mechanisms of action and the identification of drug response predictors (60). In addition, this drug is an antagonist of other receptors, such as H1, 5-HT_2C_, and M3, leading to weight gain and metabolic side effects (61) that include both glycemic dysregulation and insulin resistance (62) (see Table 1).

Clozapine may have several interactions with other drugs as it is metabolized by the hepatic cytochrome P450 (CYP) system. Clozapine is transformed into norclozapine by CYP3A4 and 1A2 and clozapine N-oxide by CYP3A4. Nevertheless, CYP2C19 is also significant at clozapine therapeutic concentration (24%) while the influence of CYP2C9 (12%) and 2D6 (6%) is more modest. Then, blood-level monitoring of clozapine may be needed when inhibitors (such as antifungals, oral contraceptives, fluvoxamine, ciprofloxacin, caffeine, and disulfiram) or inducers (such as rifampicin, omeprazole, phenytoin, phenobarbital, and tobacco smoke) of CYP1A2 and both inhibitors (such as cimetidine, erythromycin, and clarithromycin) and inducers (as carbamazepine and rifampicin) of CYP13A4 are used. It is important to note that tobacco smoking may affect clozapine metabolism through CYP1A2 induction (59).

Endocrine alterations induced in animal models by clozapine administration have been observed at doses of 1–10 mg/kg (63), 7.5 mg/kg (64), 10 mg/kg (65), and 2–20 mg/kg (66). Additionally, clozapine significantly increased leptin levels (67) due to its affinity to M3 receptors, which have been linked to decreased insulin released by β-cells (63, 68), regulated glucose homeostasis, and body weight (69, 70). Indeed, studies have shown that only olanzapine and clozapine have a substantial affinity to M3 receptors. In this sense, the overall increase in leptin levels and its association with BMI suggest that leptin acts as a negative feedback signal in the event of fat increase (63).

A potential metabolic impairment by clozapine via the hypothalamic insulin signaling pathway has been reported *in vitro*. Using mHypoE-46 and rHypoE-19 neuron cell lines, clozapine impaired insulin-induced phosphorylation of AKT (63). Although clozapine is known to inhibit 5-HT_2A_R signaling through G protein-dependent mechanisms, it differs from classic GPCR antagonists in that it also induces 5-HT_2A_R internalization and activates AKT signaling through a 5-HT_2A_R-mediated event (71). An animal model, where this drug (2.5, 5, 10 mg/kg) was applied intravenously to Wistar rats, showed an acute increase in corticosterone and glucagon levels, which explains the establishment of hyperglycemia (72).

The vasoactive intestinal peptide (VIP) of parasympathetic origin may contribute to clozapine (muscarinic M1-receptor)-induced sialorrhea, an adverse effect created by its synergistic interaction with the antipsychotic in some patients with schizophrenia (73). Therapeutic doses of clozapine may induce reproductive dysfunction through mechanisms involving ovarian mitochondrial dysfunction and oxidative stress (74), an effect explained by the impairment of the mitochondrial respiratory chain. This phenomenon is supported by a study with thirty adult female albino rats that received clozapine (20 mg/kg/day) for 28 days. It was observed that reduced complex I activity (25%) resulted in a 35% decrease in ATP and mitochondrial respiration, thus severely impairing energy production and leading to apoptosis (75).

Resistin is a biomarker of systemic inflammation and likely plays a role as a marker of cardiovascular comorbidity. A study showed that Clozapine (40 μM) inhibited resistin mRNA expression in mouse brown adipocytes (76). However, in 121 schizophrenia patients treated with clozapine (403 mg/day), high serum levels of resistin were associated with smokers in comparison with non-smokers (77).

The interaction between clozapine and its pharmacological target in leukocytes induced inflammatory alterations in cell lines, primary cultures, animal models, and humans. The principal immune alteration associated with clozapine is agranulocytosis (neutrophils <500 cells/mm^3^), the most severe form of leukopenia affecting approximately 3.9% of users, and others as neutropenia (neutrophil count <1,500 cells/mm^3^) (78). The risk of neutropenia/agranulocytosis is 0.38% approximately with monitoring and 2.5% without it (79). This was mainly observed in female patients and was directly associated with the time of clozapine use (37, 80). Clozapine by itself was not directly toxic to neutrophils or their progenitors at therapeutic concentrations (79). However, the bioactivation/oxidation of clozapine in neutrophils produced reactive and unstable clozapine metabolites, which induced toxic oxidative stress, leading to neutrophil apoptosis. Metabolites may be cytotoxic to bone marrow stroma, potentially leading to accelerated neutrophil or myelocyte precursor apoptosis (81, 82). An associated genetic susceptibility was detected in 31 patients who developed clozapine-induced agranulocytosis and 38 patients who developed neutropenia in a group of 310 clozapine users. The most significant association was found with mutation NQO2 G1541A, making it one of the candidate markers for the prediction of these adverse effects (83).

The current pharmacovigilance processes, carried out worldwide, have allowed for the identification of three uncommon cases of clozapine-induced drug reaction with eosinophilia and systemic symptoms (84). These three reported cases took place in adults older than 57 years, all of them consuming different drugs previously. Two patients were diagnosed with acute exacerbation of a chronic paranoid schizophrenia and the third presented schizoaffective disorder. In the three cases, 15–22 days into the treatment (200–400 mg/day), the blood levels of clozapine were within the toxic range, while eosinophilia, leukocytosis, and liver abnormalities were detected along with a significant increase in CRP without infection (85, 86).

The phenomenon observed in these patients was secondary to the inflammatory process leading to an increase in circulatory levels of clozapine. It is known that cytokines can inhibit the metabolism of clozapine through cytochrome 4501A2 inhibition (87).

Clozapine is also associated with changes in lymphocyte phenotype and differentiation as well as changes in cytokine secretion. Some evidence showed that clozapine primarily inhibited the expression of 5-HT_2A/2C_ on the membrane of primary T cell cultures and Jurkat and CEM cell lines (29, 72). Additionally, it is known that clozapine *in vitro* (1.5–7.5 μg/mL) inhibits Th1 differentiation by preventing the expression of transcription factor T-bet but not that of STAT-4 in T cells; clozapine also inhibited Th1 differentiation by blocking the AKT activation pathway (88). Moreover, clozapine (20 μM) promoted the *in vitro* differentiation of Treg cells and the expression of Foxp3 in splenocytes and lymph node cells from C57BL6/J mice in a model of experimental autoimmune encephalomyelitis (EAE) (15).

Regarding the effects of clozapine in MQs it has been described that clozapine increased IL-10 production and decreased IL-12 secretion in MQs after 5 days of incubation and when it is stimulated with lipopolysaccharide (LPS) for 24 h (89). Similarly, clozapine (10–100 μM) reduced nitric oxide (NO) and IL-12p40 production by LPS-stimulated bone marrow-derived macrophages (BMDM) from female C57BL/6 mice (90).

In studies carried out in animal models, similar effects to those described above in cell cultures have been observed in MQs. In a perinatal phencyclidine rat model, the administration of clozapine increased IL-6 and TNF-α with sex-specific changes (91), which can fit in the theory of “cytokine signature” observed in blood leukocytes from healthy volunteers incubated with clozapine (1 μM) (92). It has also been reported that, in Wistar rats, clozapine (45 mg/kg/day) induced myocarditis related with lymphocytic infiltrates, which induced the release of reactive oxygen species (ROS), cytokines, and TNF-α (93). Additionally, a perinatal model of 90 day-old Wistar rats prenatally treated with LPS reported that daily clozapine (10 mg/kg) significantly reduced IL-1β, TNF-α, and IL-2 levels (60, 94).

Finally, consideration should be given to the changes in the profile of circulating cytokines induced by clozapine consumption. For instance, nine patients with diagnosed schizophrenia or schizoaffective disorder, who were treated with clozapine 100–400 mg/day, showed increased risk of developing fever after the first intake, and IL-6 might play a specific role in the interaction effect between treatment duration and fever development (94, 95). Clozapine has also been shown to increase soluble IL-2 receptor (sIL-2R) and IL-6 levels (96, 97). Similarly, the adipokine resistin was associated with several acute and chronic inflammatory states and promoted the expression of TNF-α and IL-6 by human mononuclear cells (97) (see Table 2).

**Table 2:** Immunoendocrine peripheral effects induced by atypical antipsychotics.

**AAPs**	**Diseases**	**Endocrine effects**	**Immune effects**
Olanzapine	• Schizophrenia • Bipolar disorder (mixed or manic episodes)	• Lower concentrations of BNDF • Insulin-resistance • Increase in serum prolactin (only female in short term treatment) • Decrease in serum prolactin (long term treatment) • Hyperinsulinemia • Diabetic ketoacidosis • Increase in leptin • Decrease in ghrelin • Increase in postprandial ghrelin (rats) • Decrease in cortisol	• Increased levels of IL-1, IL-6, and TNF-α (mice) • Eosinophilia • Hypersensitivity syndrome • Leukopenia • Decrease in IL-6 and TNF-α production (THP-1 cells)
Clozapine	• Treatment-resistant schizophrenia • Psychosis • Depression	• Glycemic deregulation • Insulin resistance • Increase in leptin levels • Increased cholesterol concentration • Sialorrhea (secondary to VIP interaction with muscarinic receptor) • Ovarian mitochondrial dysfunction	• Increased levels of IL-10, IL-6, and TNF-α • Decreased levels of IL-12 • Reduction in NO levels • Decreased expression of 5-HT_2A/2C_ in T lymphocytes • Inhibits Th1 differentiation
Quetiapine	• Schizophrenia • Bipolar disorders • Depression • Bipolar depression • Anxiety • Delirium • Obsessive compulsive disorder	• Low incidence of hyperprolactinemia • Insulin resistance • Low levels of insulin • Hyperglycemia • High levels of glucagon • High levels of growth hormone • Low levels of T4 and free T4 • High levels of TSH • Low levels of cortisol	• Neutropenia • Leukopenia • Agranulocytosis, • Thrombocytopenia • *In vitro*: low levels of IL-2 • *In vitro*: high levels of TNF-α and IL-17 • *In vitro*: high levels of IL-4 and IL-10 • *In vitro*: low levels of IFN-γ • High plasma levels of BDNF
Asenapine	• Schizophrenia • Bipolar disorders • Bipolar disorder in children and adolescents	• Hyperinsulinemia • Variation in glucagon release • Hypoprolactinemia	• No available data
Aripiprazole	• Bipolar disorder (manic and mixed episodes) • Schizophrenia • Irritability associated ASD • Tourette syndrome • Adjunctive treatment for MDD	• Increased DNA methylation of GLUT1 • Increased fatty acid synthesis and hypertriglyceridemia • Decreased levels hyperprolactinemia	• Decreased levels of TNF-α, IL-8, IL-21, IL-13, IL-17, CXCL1, CXCL10, CCL4, IFN-γ, IL-1-β, IL-6, IL-12, IL-23, and IL-4. • Reduction of levels of PGE2, COX2, and NO. • Increase Glutathione peroxidase (GSH-Px) and Superoxide Dismutase (SOD)
Risperidone	• Schizophrenia • Schizoaffective disorder • Schizophrenia in pediatric population s (13-17 years) • Bipolar mania in pediatric population (10-17 years) • Autism-related irritability (>5 years)	• Hyperprolactinemia (human and pigtail macaques). • Decrease in testosterone levels in women and estradiol levels in both genders. • Increase in leptin and insulin levels. • Increase in TSH levels. • Decrease in adiponectin levels. • Increase in insulin levels in FVB/N line (mice). • Decrease in α-MSH, AgRP, and CART (rats). • Increase in glucagon, leptin, and ghrelin levels (rats). • Ovarian mitochondrial dysfunction (rats).	• Leukopenia • Neutropenia • Lymphopenia • Thrombocytopenia • Fever • Development of acute eosinophilic pneumonia • Elevated BDNF levels only in relapsing males • Increase in IL–6, TNF-α, and CRP levels (rats) • Decreased IL-1β, IL-6, IL-8, MIP-1β, fraktaline, TNF-α, IL-7, IL-13 IL-17a, IL-23, IL-21, IL-4, IL-10, eotaxin, and MCP-1 levels. • Increase in IL-10, IL-RA, and TNF-α levels. • Increase in Igγ chain levels. • Decrease in titers of platelet-associated antibodies titers. • Reduced platelet aggregation. • Reduction in IFN-γ production and Th1 differentiation by PBMCs.
			• Reduction in IFN-γ production by CD4 T cells. • Increased IL-10, IL-6, IL-8, and TNF-α production by MDDCs. • Decreased IP-10 and IL-12 production by MDDCs with neutrophil death and decreased IFN-γ secretion by T cells • Inhibition of adhesion, phagocytosis, and ROS in U-937 cells • Decrease in IL-6 and IL-8 and increase IL-10 production by macrophages. • Decreased Th17 cell count. • Induction of NF-κB target genes in adipocytes. • Increased TLR2 expression in T cells. • Decreased TLR4 expression in monocytes. • Up-regulation of genes in blood cells: cytokine receptors, PRRs, molecules involved in apoptosis, *BDKRB1, IGF1R*, and *CR1*. • Increased of VCAM, ICAM, E-selectin, MCP-1, and TNF-α levels in aortic tissue (rats) • Decreased IL-17, IL-2, and IL-4 secretion in acute EAE; increase splenocytes Tregs, CD4^+^ T cells and IFN-γ levels in chronic EAE (mice) • Decrease IL-12 and increase IL-10 production; reduction of IFN-γ, IL-17, and increase IL-10 production by T cells (mice). • Increased NO levels and apoptosis; decreased Bcl/BAX, IL-10 production and increase IL-1, IL-6, TNF-α and IFN-γ in RAW 264.7 line (mice).
Paliperidone	• Schizophrenia • Schizoaffective disorder • Schizophrenia in pediatric population (12-17 years)	• Hyperprolactinemia • Elevated insulin levels.	• Leukopenia • Neutropenia • Lymphopenia • Agranulocytosis • Increase in BDNF levels • Enrichment of NF-κB pathways • Decrease in cell survival (U-937 cell line)
Iloperidone (see Supplementary Material section)	• Acute phase of schizophrenia in adults • Stabilization phase of schizophrenia	• Hypoprolactinemia • Hyperprolactinemia with galactorrhea	• No available data
Ziprasidone	• Schizophrenia • Bipolar disorders	• Hyperprolactinemia with galactorrhea • Hypocortisolemia	• Agranulocytosis • Low levels of IL-10 • It induced allergic responses: high levels of IgG and complement proteins C3 and C4 • *In vitro*: high levels of NO and ROS • *In vitro*: high levels of IL-1, IL-6, TNF-α, and IFN-γ
Lurasidone(see Supplementary Material section)	• Schizophrenia • Depression associated with bipolar disorder	• Decrease in levels triglyceride levels • Increase in HDL cholesterol	• Decrease in C-reactive protein (CRP) • Leukopenia • Thrombocytopenia

## Risperidone

Risperidone was the second AAP approved by the FDA and is among the most prescribed worldwide (98). Its use was authorized for the treatment of schizophrenia in 1993; it was approved to treat acute manic or mixed episodes of bipolar I disorder as monotherapy or adjunctive drug in 2003 and autism-related irritability in 2006 (99). There are also many varied non-FDA approved uses for risperidone, such as Tourette syndrome (100), major depressive disorder (MDD) (101), anorexia nervosa (102), dementia (103), borderline personality disorder (104), Parkinson's disease psychosis (105), posttraumatic stress disorder (106), and some other psychiatric conditions (107). This drug has liver metabolism; it mainly undergoes 9-hydroxylation that produces active 9-hydroxy-risperidone (OH-RIS) metabolite by CYP2D6 and CYP3A4 to a lesser extent (107). The available formulations of risperidone in the market are oral solution formulation, oral disintegrating tablets, and long-acting injectable (LAI) formulation (108).

The presumed action mechanism of risperidone is associated with the combination of 5-HT_2A_ agonist and D2 antagonist effects with a strong binding affinity for the first one (see Table 1) (109). This drug is also active as an antagonist for other receptors with a lower affinity, such as dopamine D3 and D4, serotonin 5-HT_6_, 5-HT_7_, and α-1 and α-2 adrenergic. Risperidone acts as agonist on 5-HT_2C_ serotoninergic receptors and H1 histaminergic receptors (43).

Risperidone consumption has demonstrated to generate hormone alterations in human and animal models. Reports on this AP refer to hormones related with glucose metabolism, adipokines, appetite, and those linked to the adrenal, and gonadal axes, among others. One of the main effects associated with risperidone consumption is elevated PRL levels in patients (110), having a significant incidence of HPRL compared with other APs (111–113). In human and adult studies, the drug caused significant PRL elevation after 44 days of treatment (1–6 mg/day) in 27 of 37 schizophrenic patients, while in a 1 year follow-up (1–6 mg/day) 6 out of 20 patients reported HPRL with decreased PRL, without reaching baseline levels (114). These results were similar to those obtained by Perez-Islas et al., whose report showed that 90% of the men and 87% of the women in the study had PRL levels above the reference range 3 months into the treatment (unspecified dose). These levels were still elevated in 70% of the subjects at 1 year of follow-up with a tendency to decrease (115).

During an acute follow-up (4 weeks), the patients with an increase of more than 20% in PRL levels had a better chance of responding to risperidone (116). However, the chronic consumption gave more information on PRL concentration and its effects; the reports proved that risperidone is associated with chronic HPRL (117). Female patients presented a significant incidence of HPRL as compared with their male counterparts (118). Elevated PRL by risperidone consumption could be associated to higher concentrations of osteocalcin in both genders (119), breast symptoms, discomfort, menstrual changes, and erectile dysfunction (120). In fact, patients who consumed risperidone and showed menstrual disorders had a significant increase in serum PRL levels, showing a correlation between the incidence of elevated PRL and menstrual disorders (121). Other organic and rare alterations, in adult and/or children patients, as granulomatous mastitis (122), amenorrhea (123), galactorrhea (124), acute pancreatitis (125), and pituitary adenoma (126) have also been associated with risperidone consumption.

The reports on PRL levels in children and adolescent patients have shown that these populations present elevated HPRL, which has been reported in 44.9% of autism spectrum disorder (ASD) patients as unrelated to the duration of risperidone treatment (0.25–5 mg/kg, 1.03–158.03 months) (127). Still, there is evidence exposing the possible relation between plasma metabolite levels of risperidone and elevated PRL concentrations (128). Patients from the same population presented an increase in serum PRL during 3 months of follow-up (0.5–4 mg/day); gender, pubertal status, risperidone dosage, psychiatry diagnosis, and personal/family history of autoimmune diseases also affected PRL elevation during treatment (129). Similarly, a meta-analysis reported that pediatric patients treated with risperidone (4–6 mg/day) were found to experience the most significant increase in PRL, followed by patients treated with 1–3 mg/day of risperidone compared with other APs at a different dose (130). In fact, the occurrence of HPRL in this population has been associated to the presence of the C allele of the rs6318 single nucleotide polymorphism (SNP) of the *HTR2C* gene (0.25–6 mg/day, 0.1–143 months) (131). In addition, HPRL during treatment with risperidone/paliperidone in schizophrenic patients showed an association with rs40184 and rs3863145 variants in *SLC6A3* gene of blood leukocyte DNA (132). All the studies that showed elevated PRL levels in the pediatric population are in accordance with a meta-analysis that presented a relation between risperidone treatment and high PRL (130). The possible mechanism by which risperidone causes HPRL is associated with the transcriptional upregulation of neuropeptide Y (NPY) secreted by the arcuate hypothalamic nucleus due to the high affinity of risperidone to 5-HT_2A_ receptors. NPY inhibits tyrosine hydroxylase expression in the paraventricular nucleus and thus reduces DA synthesis, which in turn would diminish the inhibition of PRL expression induced by DA. The reduction in DA would cause the overexpression of PRL in the pituitary and ultimately induce HPRL (133).

Studies have shown that, in addition to PRL increase, other hormone profiles such as estradiol, testosterone, leptin, adiponectin, and insulin could be altered during risperidone treatment. The acute consumption of this drug (2–4 mg/Kg) decreased testosterone and estradiol levels in female patients after a 6-week treatment (134), and the same decrease in estradiol levels was reported in male patients with schizophrenia during 1 year of treatment (2–6 mg/day) (135), although other studies showed that risperidone consumption did not alter the testosterone or estradiol levels in male or female patients (136, 137). Although the mechanism is not clear, risperidone could affect estradiol and testosterone levels by a direct effect on the hypothalamic-hypophysis-gonadal axis, decreasing hormone production (135).

The concentration of leptin has also been proven to increase by 60% in psychotic patients after 4 weeks of risperidone consumption (4–8 mg/day) (138). Other studies showed that the leptin levels of schizophrenic patients with risperidone consumption were higher than those of healthy controls (139, 140). Similarly, there was an increase in leptin among ASD patients during at least 12 months of treatment (0.25–1 mg/day) (141). In treatment-naïve children and adolescents, leptin increased after 3 and 6 months of treatment (unspecified dose) when compared with baseline (142). However, a 5-month treatment (6.1 ± 1.8 mg/day) yielded no changes in leptin concentrations when compared with baseline (143); this data is supported by a meta-analysis that found no significant changes in leptin after risperidone treatment (63). The serum leptin elevation is attributed to weight gain rather than the direct effect of risperidone on leptin metabolism. This hormone is secreted by adipocytes and it is proportional to the mass of stored fat, so the elevation in blood of patients with antipsychotic-induced weight gain could be the result of the increased weight itself (144).

Adiponectin is a molecule with conflicting results on the effect of risperidone consumption. Schizophrenia patients treated with risperidone (unspecified dose, 50.1 ± 82.4 months) reduced plasma levels of adiponectin when compared with healthy subjects (145), and medication-free children showed a decrease in adiponectin levels after 16 weeks of treatment (3–91 months) (146). However, these data do not match with the results of two meta-analysis that reported no association between risperidone treatment and low adiponectin (147, 148).

According with most reports, risperidone consumption increases insulin levels in blood. The treatment with LAI-risperidone (38 ± 2 mg/15 days) for 18 ± 1.6 months showed higher insulin concentrations in patients compared with the control group (140). Children studies show that the treatment for at least 12 months (0.25–1 mg/day) increased insulin levels in ASD patients (141). Similar results were obtained in medication-free children (3–91 months) after 16 weeks of treatment (146).

The changes in hormone levels related with risperidone treatment are also evident in animal models. In a pigtail macaque model, PRL was higher at low (0.025 mg/Kg) and high doses (0.05 mg/Kg) of risperidone during 4-month consumption, with a gradual decline until reaching placebo levels in the post-drug phase (149). In an animal model, male Wistar rats showed reduced α-MSH, agouti-related protein (AgRP), and cocaine- and amphetamine-regulated transcript (CART) concentrations and increased leptin levels vs. the vehicle group after 4 weeks of treatment (2 mg/kg/day) (150). In addition, female Sprague-Dawley rats with depot risperidone exhibited higher glucagon levels (20 mg/day), while daily risperidone (40 mg) increased leptin and ghrelin levels at 4 and 6 weeks (151). In rat ovarian theca cells, risperidone inhibited mitochondrial bioenergetics and steroidogenesis by reducing ATP content (0.1–100 μM, 24 h) and the production of progesterone and androstenedione (1–37 μM, 24 h) (151). Finally, mouse models have reported plasma insulin increased over 8-fold in FVB/N mice 3 h after consumption of risperidone (152).

The adverse effects, involving immune alterations, caused by risperidone have been widely studied. In the immune system, this drug alters leukocyte numbers and levels of humoral inflammatory molecules, and it directly effects the phenotype and function of leukocytes (16, 153, 154).

Risperidone does not require regular clinical monitoring of white blood cell (WBC) count; however, anecdotal evidence has shown that it could modify and reduce the leukocyte count. Different risperidone doses (2–4 mg/day) and the combined treatment of risperidone/paliperidone (2 mg/day/100 mg) caused leukopenia with neutropenia (155) or lymphopenia (153, 156–158) as well as fever (159). Other leukocyte alterations have been described. A case report showed the development of thrombocytopenia in a male paranoid schizophrenia patient with risperidone treatment (4 mg/day) (160). Risperidone has demonstrated the association between its consumption (3 mg/day) and the development of acute eosinophilic pneumonia (AEP) in a male patient under a 6-month treatment (161). All count alterations and developed diseases improved after discontinuing the drug. Nevertheless, there are reported cases of neutropenia induced by risperidone but the incidence rate of cytopenia alterations seems to be very low (162).

The reports that analyze the effect of risperidone consumption on soluble molecules with immune function are very diverse. Even though CRP levels did not show changes in schizophrenic patients during risperidone treatment vs. healthy volunteers (163), there is evidence showing the relationship between elevated CRP and the effects on risperidone metabolism. A case report demonstrated that two females who had consumed risperidone during acute inflammation indicated by elevated CRP exhibited an increase in dose-related serum concentrations of risperidone up to the therapeutic concentration (164, 165). Furthermore, high CRP vs. common CRP values increased risperidone and OH-RIS serum levels by 58.4 and 20%, respectively, in patients with risperidone consumption (166). Contrastingly, other reports showed no correlation between CRP levels and affected risperidone levels in serum concentration (95).

The studies of changes in BDNF levels during treatment are controversial; only relapse schizophrenic males patients showed elevated BDNF after 4 weeks of risperidone consumption (3–6 mg/day), a result that suggests gender should be considered when choosing the pharmacological treatment (167–169). However, other studies reported no alteration whatsoever after risperidone consumption (170, 171).

Cytokines, chemokines, and immunoglobulins (Ig) are inflammatory molecules that have been measured during risperidone treatment and results show changes in blood levels in some of them. In an animal model, risperidone decreased and normalized the plasma levels of IL-6 and TNF-α in n-3 fatty-acid deficient rats when compared with elevated levels of n-3 fatty-acid adequate rats after 40 days of treatment (3 mg/kg/day) (172).

In patients with schizophrenia, the measurement of these molecules in blood has proven the immunomodulatory effect of risperidone at different times of consumption. A 3-month treatment with risperidone (1–6 mg/day) showed significant decreases in serum levels of IL-8, macrophage inflammatory protein (MIP)-1β, fractalkine, TNF-α, IL-7, IL 13, IL-17a, IL-23, and IL-21 (173). The same effect in TNF-α was observed at 40 days of treatment (unspecified doses) with increased IL-10 serum levels in patients with risperidone or clozapine consumption (174). Elevated levels of Interleukin-1 receptor antagonist (IL-1RA) and IL-10 have also been reported after 6 weeks of treatment (unspecified doses) (175) as well as significant decreases in IL-6, IL-10, TNF-α, and IL-4 after 10 weeks of risperidone consumption (4 ± 1.8 mg/day) (176). During 6 months of risperidone treatment (2–6 mg/day), TNF-α levels increased compared with baseline while IL-1β and IL-6 decreased at 1 month and then gradually increased at the end of the follow-up (154). In ASD patients, eotaxin and monocyte chemoattractant protein-1 (MCP-1) levels significantly decreased after 8 weeks of treatment (0.5–1.5 mg/day) (177). Regarding Igs, a report showed that a 4-week treatment with risperidone increased Igγ (IgG) chain levels significantly when compared with baseline (178). Another report showed that from 17 schizophrenic children with high blood titers of platelet-associated antibodies (PAA) only two became PAA-negative following 3 years of treatment. Most of the reports above show evidence that risperidone could reduce the production of the pro-inflammatory molecules caused by psychiatric conditions and support an anti-inflammatory response.

When talking about phenotype and function alterations in immune cells, it seems that risperidone causes significant changes such as a shift in cytokine secretion, cell differentiation, adhesion and phagocytic functions, receptor expression on leukocytes, and gene expression. Firstly, this drug reduces ATP-induced platelet aggregation when platelet-rich plasma of healthy donors is incubated *in vitro* with risperidone (65 ng/mL) for 30 min (179). An *in vitro* assay showed that activated peripheral blood mononuclear cell (PBMC) from healthy adults incubated with risperidone (10^−7^ M, 3–5 days) reduced IFN-γ production and inhibited AKT phosphorylation and T-bet expression, causing reduced Th1 differentiation. During chronic treatment (10^−7^ M, 28 days), risperidone reduced IFN-γ released by CD4^+^ T cell subpopulation (180). Similarly, this drug affected cytokine and chemokine production of activated mature monocyte-derived dendritic cells (DCs) of healthy adults, increasing IL-10, IL-6, IL-8, and TNF-α levels and decreasing interferon γ-inducible protein−10 (IP-10) and IL-12 levels (10^−7^-10^−5^ M, 3 days). These changes in mature DCs produced a reduction in IFN-γ secretion by activated T cells, causing Th1 suppression and leading to neutrophil death (15, 17).

The increase in IL-10 levels and/or the decrease in IFN-γ production by activated PBMC with risperidone treatment were reproducible in other reports (16). Risperidone also inhibited the adhesion, phagocytosis, and ROS production by activated U937 cells (10^−5^-10^−4^ M), decreased IL-6, IL-8, and IL-12, and increased IL-10 production in healthy, stimulated human MQs *in vitro* (10^−6^-10^−5^ M). This effect could support the inhibition of Th1 differentiation (181, 182), although some evidence proposes that this drug suppresses inflammatory (M1 MQs, Th1 lymphocytes) and anti-inflammatory (Th2 lymphocytes, Treg) responses (90).

In the blood of schizophrenic patients, the treatment with risperidone (2–6 mg/day) for 4 weeks showed a decrease in the number of Th17 cells (183). *In vitro*, differentiated human adipocytes incubated with risperidone (100 ng/ml, 11 days) induced transcription factor NF-κB target genes of IL-1β and IL-8 molecules (184). In schizophrenic patients, risperidone (8 weeks, 2–6 mg/day) modifies the expression of toll-like receptors (TLR), while monocytes CD14^+^, CD3^+^CD4^+^Foxp3^+^ T, and CD3^+^CD4^+^CD25^+^ T cells increased TLR2 expression, and CD14^+^ monocytes decreased TLR4 expression (185). Effects on gene expression have been reported; in blood cells of first-episode psychosis patients, risperidone (unspecified doses, 20 days) was associated to the up-regulation of 11 immune system genes, including cytokines and cytokine receptors (*SPP1, IL1R1, IL1R2*), pattern recognition molecules *(TLR1, TLR2, TLR6*, dectin-1/*CLEC7a*), molecules involved in apoptosis (FAS), and *BDKRB1, IGF1R*, and *CR1* (186).

In animal models, a 3-week treatment with risperidone (1.25 mg/Kg/day) in diabetic Wistar rats showed that this drug altered the vascular function by the significant up-regulation of vascular cell adhesion molecule-1 (VCAM-1), intercellular adhesion molecule-1 (ICAM-1), E-selectin, and MCP-1 and TNF-α in aortic tissue homogenate (187). In an experimental autoimmune encephalitis (EAE) model with C57BL/6 mice, risperidone (3 mg/kg/day) reduced the severity of the disease in a dose-dependent manner and down-regulated IL-17a, IL-2, and IL-4 secretion by splenocytes at peak disease (day 15). During chronic EAE phase, risperidone significantly increased the number of splenocytes, Tregs, and CD4^+^ T cells and increased IFN-γ levels, showing that T cells responded differently to risperidone during the acute and chronic phases of EAE. In addition, activated BMDM of treated mice decreased IL-12 levels but increased IL-10 concentration. These cells modified T cell activation reducing IFN-γ and IL-17 production and enhancing IL-10 levels (188). In RAW 264.7, a macrophage mice line, risperidone activated these cells (20–40 μM for 24, 48, and 72 h) and increased nitric oxide (NO) levels (30–40 μM) as well as apoptosis events by modulating levels of caspases 8 and 3 (20–40 μM at 72 h). The drug also reduced Bcl-2/BAX gene expression ratio (24 h) and, contrary to the above data, increased IL-1, IL-6, TNF-α, and IFN-γ and decreased IL-10 production in a dose-dependent manner. Results of the RAW 264.7 line could show that the continued activation of MQs likely contributes to the development of endocrine disturbances caused by risperidone (91) (see Table 2).

## Olanzapine

Olanzapine (2-methyl-4-(4-methyl-1-piperazinyl)-10H-thieno[2,3-b][1,5]benzodiazepine) belongs to the thienobenzodiazepine class and is structurally similar to clozapine (see Figure 1) (189). It was authorized for the treatment of schizophrenia in 1996 and bipolar I acute manic or mixed episodes in 2000 (43, 109, 190). There are also many varied non-FDA-approved uses for quetiapine, such as dementia-related behavioral problems, bipolar depression, psychotic depression, SSRI-resistant major depression, personality disorders, post-traumatic stress disorder, and Tourette syndrome in children and adolescents (191). Olanzapine is metabolized in the liver by direct glucuronidation and cytochrome P450 (CYP) oxidation and generates two metabolites, 10-N-glucuronide and 4'-N-desmethylolanzapine, which lack pharmacological activity (192).

The therapeutic effect of this drug is associated to the antagonism of D2, D3, 5HT_2a_, and 5-HT_2c_ receptors, although it exhibits an antagonist effect on other receptors such as 5-HT_1C_, 5-HT_6_, 5-HT_7_, α-1A, α-2A, H1, M1, M3, and 5-HT_1A_ (see Table 1) (31–35).

This drug has been associated with the decrease in cell counts. There are few reports that evidence leukopenia is induced by olanzapine consumption (2.5–10 mg/day) during the first 35 days of treatment (193, 194). This phenomenon is associated with the covalent bonding between neutrophils and a reactive nitrenium ion, the oxidized form of olanzapine. It has been proposed that this reactive metabolite is responsible for the effect in neutrophils (195). Other studies have reported that this drug is associated with a decrease in eosinophils. Three reported cases showed that males with schizophrenia developed eosinophilia during olanzapine treatment (10–20 mg); in all cases, the problem was solved suspending the medication (193, 194, 196). This drug also modifies receptor expression; according to a report, 30 first-episode psychotic patients treated with olanzapine (15–25 mg/day) for 30 days showed a decrease in D1, D2, 5-HT_2A_, and transforming growth factor (TGF)-β mRNA expression in PBMC as well as an increase in IL-6, IL-1β, and TGF-β blood levels (197).

The increase in PRL caused by the consumption of most of the AAPs is also observed in the consumption of olanzapine. However, the evidence of this effect is contradictory since there is also proof of the decrease during the consumption of this drug. It has been reported that according to the period of consumption, the effects on PRL blood levels change (111, 198, 199): The consumption during short periods (<2 weeks) or the intake of a single dose does not alter PRL levels (111, 200).

There are two case reports of women aged 29 and 49 years with bipolar affective disorder and delusional disorder who exhibited HPRL associated with olanzapine consumption during 24 weeks (5–20 mg/day) (201). Most reports evidence the increase of this hormone: A study with 72 patients (33 women and 39 men) who were administered olanzapine (10–20 mg/day) for 3 weeks or more showed an increase in PRL levels only in female patients (202). Similar effects were detected in 49 schizophrenia patients (24 women and 25 men) treated with 15–30 mg/day of olanzapine for 4 weeks (203) and those patients (27) with chronic consumption (10–15 mg/day, 8 years) (204). In fact, a study with healthy volunteers showed that participants with no psychopathology who received one dose of olanzapine (10 mg) exhibited an increase in PRL levels (58). On the other hand, other studies showed the decrease of PRL after olanzapine consumption. In a study with 22 participants, PRL levels were reduced only in women with schizophrenia or schizoaffective disorder at 6 and 12 months of treatment (5–20 mg/day), while levels in men showed no difference (198). The decrease in PRL levels was also reported in 37 first-episode psychosis patients who consumed olanzapine (unspecified dose) for 1 year (115). The PRL elevation is associated with the interaction between olanzapine and D2 receptors on lactotroph cells. This phenomenon hampers the interaction between DA and its receptor, so DA cannot inhibit PRL production. The alterations depend on gender, genetic predisposition, dose, and time of consumption (115, 190, 201, 205).

Interestingly, the chronic administration of olanzapine is associated with the development of hyperinsulinemia and insulin resistance; the decrease in insulin sensitivity was reported in 29 healthy individuals after 10 days of olanzapine treatment (10 mg/day) (206). The increase in fasting insulin was reported in 25 schizophrenic patients with olanzapine consumption (5–20 mg/day) for 13 weeks (207). It has been postulated that alterations in insulin synthesis may be due to the stimulation of M3 receptors in β-pancreatic cells (70, 208).

It has also been described that olanzapine is associated with an increase in leptin blood levels (63), although there is little evidence that shows no changes in leptin blood levels during treatment. The increase in this hormone was shown in 18 schizophrenic male patients after 9 months of olanzapine consumption (5–20 mg/day) (209). Another study reported that 23 schizophrenic patients showed the same effect after 8 weeks of olanzapine treatment, and the increase in leptin concentration was correlated with elevated IL-1 receptor antagonist (IL-1ra) serum levels (210). In fact, the report of Tsuneyama and cols. described that the increase in leptin levels was observed only in schizophrenic female participants treated with olanzapine for 1 year (12 male and 19 female) (211). However, 12 schizophrenic patients exhibited no changes in appetite or leptin concentrations after a 5-month treatment (mean: 25 mg/day) (143). Although the mechanism by which this drug affects leptin secretion is unclear, the effect could be secondary to the interaction between the drug and H1 receptors on the hypothalamus and nucleus accumbens. Additionally, the genetic predisposition is crucial for the development of alterations associated with leptin function (212).

The data of reports on the olanzapine effect in ghrelin levels exhibit contradictions in the conclusions; some studies in schizophrenic patients show that this drug reduces ghrelin concentration after a 6-week treatment (213) and chronic consumption (8.3 ± 7.5 years, 10–20 mg/day) (214). In contrast, no changes in ghrelin levels or appetite were shown in 13 patients with schizophrenia treated with olanzapine for 5 months (143). However, an animal model with Wistar rats showed that the acute consumption of this drug (1 mg/kg) increased the concentration of postprandial ghrelin compared to controls (215). The mechanism of ghrelin alterations is not clear but these changes are associated with leptin resistance. It has been proposed that olanzapine exhibits a direct effect on hypothalamic neurocircuits that regulate ghrelin synthesis, causing an altered leptin/ghrelin ratio (212). There is only one report that measured cortisol levels during olanzapine treatment: Hahn and cols. reported that healthy individuals who received a single dose of olanzapine (10 mg) exhibited a decrease in cortisol serum levels compared with baseline (58).

Other immune alterations associated with olanzapine consumption are the modulation of cytokine secretion and production, depending on the consumption time of the drug (216). There are *in vitro* studies in PBMC of healthy individuals and THP-1 line (10^−4^ M for 72 h) that demonstrate a reduction in mRNA expression of IL-1β, IL-6, IL-10, and TNF-α, IL-6, TNF-α, and IL-10. Similarly, stimulation in THP-1 cells resulted in a significant decrease in the expression and secretion of IL-1β and TNF-α (217). Other studies in schizophrenic patients with prolonged consumption reported changes in cytokine levels. After a 24-month olanzapine treatment (unspecified dose), 95 schizophrenic patients with metabolic syndrome showed lower concentrations of BDNF (*P* < 0.012) and higher values of TNF-α as compared to 121 patients only diagnosed with schizophrenia (218). Also, out of 28 patients with chronic olanzapine consumption (unspecified dose) 14 were insulin-resistant and had a higher concentration of TNF-α, IL-6, IL-1β, and IL-8 with a positive correlation between these values and insulin resistance (210). Similarly, female Sprague-Dawley rats and female BALB/c mice, after 8 weeks of treatment (10 mg/kg/day), exhibited a significant increase in TNF-α, IL-6, IL-1β, and IL-8 levels, in addition to insulin resistance (219). Some evidence suggests that the effect of olanzapine under cytokine secretion is gender-dependent; female Sprague-Dawley rats given olanzapine (low dose 2 mg/day; high dose 4 mg/day) for 3 weeks showed increased IL-8 levels, while males showed TNF-α concentration during low dose consumption, proving a gender-dependent difference. Also, compared with those of the control group, IL-6 levels were reduced in males after both doses of olanzapine while IL-1β concentration was reduced in females after a low dose (208).

Olanzapine can also modulate TLR expression in leukocytes. A study that evaluated 24 schizophrenic patients after 8 weeks of treatment (10–25 mg/day) exhibited that this drug increased TLR2 expression and decreased TLR4 and TLR5 in CD14^+^ monocytes. Treg and Tact cells reduced TLR2 and increased TLR5 expression (186). In 23 patients diagnosed with schizophrenia and treated with olanzapine for 8 weeks, IL-1RA was overexpressed, which correlated with the increase in leptin (210) (see Table 2).

## Quetiapine

Quetiapine is an AAP derived from benzothiazepine (220) (see Figure 1) and used for the treatment of psychotic symptoms in a wide range of disorders. Its use was authorized for the treatment of schizophrenia in 1997; it was authorized to treat unipolar and bipolar disorders in 2003 and bipolar depression in 2006. There are also many varied non-FDA-approved uses for quetiapine, such as anxiety, delirium, obsessive compulsive disorder, and the combined treatment of major depressive disorder (MDD) with antidepressants (221). Quetiapine metabolism, which comprises several steps as sulfoxidation, *N*- and *O*- dealkylation, and 7-hydroxilation by the CYP3A of the cytochrome P-450 system, produces *N*-desalkylquetiapine (norquetiapine), an active metabolite of quetiapine (222). Quetiapine is considered a multifunctional drug since it acts on three systems: dopaminergic, serotonergic, and noradrenergic (223). It shows high affinity for serotonin (5-HT) and DA type-2 receptors, slightly higher for the serotonergic than the dopaminergic. Contrastingly, lower affinity has been reported for type-1 receptors of both systems: D1 and 5-HT_1A_. Moreover, it is known that quetiapine also has affinity for histaminergic (H_1_) and adrenergic systems (α-_1_ and α-_2_) (see Table 1) (38).

It is well-known that most SGAs produce HPRL; however, quetiapine is considered among the safest medications due to its lower incidence of HPRL. Such properties have been associated to its lower affinity to Sackett et al. (149) and fast dissociation rate from DA receptors (224). In schizophrenia patients with sexual dysfunction, the treatment usually begins with PRL-sparing antipsychotics, switching to quetiapine in a second phase (225). In fact, quetiapine has been reported to revert HPRL in 175 patients with schizophrenia after a 2-week treatment (300–700 mg/day) (226).

On the other hand, several studies have shown the adverse endocrine effects produced by the administration of APs. In patients (12) with schizophrenia, quetiapine consumption induced significant insulin resistance. Nine months after administration, it led to a reduction in insulin sensitivity, as a result of a deficient secretion of insulin by the β-pancreatic cells (227). After a 10-month treatment with quetiapine, 16 youths (9–18 years) showed decreased levels of insulin associated with an impairment in β-pancreatic cell function (228). In mice, quetiapine administration (10 mg/kg) induced an increase in plasma levels of glucose but not in insulin, suggesting an insulin-blocker role of quetiapine in the insulin-secretory compensation mechanism (152), a finding supported by *in vitro* studies (229). McNamara et al., demonstrated that stearoyl-CoA desaturase-1 (scd-1), an enzyme involved in triglyceride biosynthesis and whose up-regulation showed a positive correlation with quetiapine consumption, could be involved in both sensitivity and insulin resistance (230). Moreover, the higher activity of scd-1 has been suggested as a risk factor for diabetes in humans (231), which reinforces the link between scd-1 and the adverse effects of quetiapine consumption. On the other hand, studies in rats have suggested that quetiapine-induced hyperglycemia was produced by increased levels of glucagon and suppressed glucagon-like peptide-1 (GLP-1) more than insulin resistance (66). Disturbances in glucagon and GLP-1 caused serious alterations in glucose metabolism because of stimulated hepatic glucose production (232).

In healthy volunteers aged 18–21 years, a dose of 150 mg/day of quetiapine was tested and the results showed an increase in PRL and growth hormone (GH) after 60 and 210 min of administration, respectively; in contrast, cortisol showed a decrease at 240 min and no changes were observed in ACTH (233). The alterations observed in GH levels by quetiapine consumption might be attributed to the high affinity and antagonism between the drug and H1 receptors. It should be noted that PRL in healthy volunteers showed a different behavior than that observed in patients, but, importantly, the sampling time used in healthy volunteers was very short. However, data in healthy volunteers are controversial, since other reports have shown no effects on PRL, but on ACTH due to the consumption of quetiapine in short sampling periods (234). Disturbances in ACTH and cortisol could be due to alterations in functioning of hypothalamus-pituitary-adrenal (HPA) axis in psychiatric patients, but the exact mechanism remains unclear. Moreover, quetiapine has revealed affectations in the levels of thyroxine (T4) and thyroid-stimulating hormone (TSH) (cases reports) with doses of 300–350 mg/day which induced a decrease in T4 and free T4, whereas TSH was increased (235).

Although the precise mechanism by which quetiapine induces adverse endocrine effects is not fully clear yet, some studies have focused their efforts on shedding light on this issue; nevertheless, more works are required to clarify this point.

Quetiapine consumption also affects the immune system. In patients with schizophrenia, quetiapine (600–1,200 mg/day, case reports) is associated with neutropenia, leukopenia (236–239), agranulocytosis, and thrombocytopenia (240). The mechanism by which quetiapine causes these adverse effects is still unclear, but some authors have proposed that this drug acts directly as a cytotoxic agent on immune cells, thus producing cell death; additionally, some products of quetiapine oxidation could induce apoptosis by oxidative stress (241). Other authors have suggested a bone marrow depression by quetiapine consumption, which could be produced by an inhibitory effect on leukopoiesis. It has even been proposed that quetiapine may act as a hapten, inducing antibody formation, complement activation, and cell death (237).

Studies *in vitro* have demonstrated the capability of quetiapine to alter the levels of some cytokines (242). Himmerich et al. demonstrated that this drug reduced IL-2 levels in whole blood cells, whereas it increased the levels of TNF-α and IL-17 (243). In PBMC cultures (LPS-stimulated) from patients with schizophrenia, quetiapine raised the levels of anti-inflammatory cytokines (IL-4 and IL-10) and lowered the pro-inflammatory ones (IFN-γ) (181). The anti-inflammatory properties of quetiapine may be explained by its capacity to suppress the NF-κB pathway activation. Quetiapine not only inhibited the expression NF-κB but also reverted its translocation from the cytosol to the nucleus, thus affecting its activation as well. These properties could explain quetiapine effects on cytokine expression (244) and have led experts to consider it a therapeutic alternative in some neuroinflammatory diseases.

Neurotrophins (NTs) are a group of neural growth factors that regulate survival, maintenance, cell differentiation, and synaptic plasticity in the CNS. But, their activity is not limited to the CNS: Cells of the immune system also express both NTs and their receptors (245), which in turn strongly contributes to the connection between neuronal dysregulation and inflammation (246). BDNF has been considered a potential biomarker of psychiatric disorders (247, 248). In patients with first episode psychosis, serum BDNF levels were increased after a 12-week treatment with quetiapine (200 or 400 mg/day). This rise in BDNF showed a positive correlation with the clinical improvement of patients, suggesting an indirect neurotrophic role of quetiapine through BDNF (249) (see Table 2).

## Ziprasidone

Ziprasidone is a psychotropic agent commonly used in the treatment of schizophrenia (250) and bipolar disorder (251, 252) since its approval by the FDA in 2001. It is a benzisothiazolyl-3-yl-piperazine-type AAP (see Figure 1) with potent pharmacological antagonism to 5-HT_2A_ and D2 receptors. However, it also acts on H1, M1, α1 and α2 receptors with less affinity (253, 254). The high affinity of ziprasidone to 5-HT_2A_ as compared to D2 is an important characteristic of this drug. However, the pharmacological antagonism of ziprasidone toward D2 makes a lot of sense considering its antipsychotic effects, whereas the role of 5-HT_2A_ receptors is still unclear (see Table 1). Still, it has been proposed that the antagonism against 5-HT_2A_ stimulates the activity of DA in mesocortical pathways (52). Ziprasidone is metabolized almost fully, excreting only 5 % of the original drug intact. Aldehyde oxidase and cytochrome CYPA34 are the two main pathways by which ziprasidone is metabolized (255).

Ziprasidone slightly disturbs PRL levels and causes low extrapyramidal effects (256). There is a case study that reported elevated PRL levels after 9 days of ziprasidone administration (80 mg/day) (257, 258). Moreover, other studies have shown that ziprasidone suppresses the activity of the HPA-axis (*n* = 11, healthy volunteers; 40 mg/day), reducing the levels of nocturnal cortisol excretion, likely due to its antagonism toward H1 and α1 adrenergic receptors (259). Studies on the adverse effects produced by ziprasidone are scarce and more research is needed.

On the other hand, the immune alterations caused by ziprasidone consumption are few. There is no sufficient evidence supporting ziprasidone causes neutropenia, but there is a case report of agranulocytosis (120 mg/day); however, this effect was attributed to a combined activity of ziprasidone and mirtazapine (260). *In vitro* studies have shown that ziprasidone and its metabolites have cytotoxic, cytostatic, and genotoxic effects on peripheral blood lymphocyte cultures, causing a reduction in mitotic, proliferation, and nuclear division indexes (261).

In RAW macrophage cell line cultures, ziprasidone can induce inflammatory response. RAW cells exposed to ziprasidone (75 ng/L) showed increased levels of NO and ROS; moreover, they showed significantly higher levels of IL-1, IL-6, TNF-α, and IFN-γ but reduced levels of IL-10 (262). Several case reports have shown that ziprasidone induced allergic responses, such as Kounis syndrome (20 mg; IM) (263), pedal edema (80 mg/day) (264), urticaria, and angioedema (120 mg/day) (265). Little is known about the adverse effects of ziprasidone, but some studies have demonstrated minor effects in the endocrine system. On the other hand, special attention should be paid to the allergic response observed after ziprasidone administration, which can be explained by the high levels of IgE and the complement proteins C3 and C4 observed in patients (264). However, it is still unclear how ziprasidone induces this response (see Table 2).

## Aripiprazole

Aripiprazole acts as a stabilizer of the dopamine-serotonin system. Its use was authorized for the treatment of schizophrenia in 2002 (266); in 2006 it was approved to treat bipolar disorder (mania or mixed episodes) (267), and major depressive disorder (as adjunctive drug) (268). In 2009 it was finally approved for the treatment of autism-related irritability (269). There are also non-FDA-approved uses for this drug such as Tourette syndrome and substance abuse disorders (270–273). Aripiprazole is metabolized in the liver by cytochrome P450, CYP2D6, and CYP3A4 by dehydrogenation, hydroxylation, and N-dealkylation. Its active metabolite, dehydro-aripiprazole, represents around 40% of the parent drug levels in plasma (274, 275). Despite the use of SGAs, this drug has several advantages for the treatment of multiple mood disorders, even if its consumption affects patients' metabolism (276–278).

Aripiprazole is a quinolinone derivate (see Figure 1); its pharmacological activity is based on its activity as a partial agonist of D2 and 5-HT_1A_ receptors and as an antagonist of 5HT_2A_. Furthermore, aripiprazole exhibits a moderate affinity to α1 adrenergic and histaminergic H1 receptors. When compared to other typical and atypical APs, aripiprazole has a higher affinity to both states of D2 receptors (see Table 1) (40–42).

There are few reports of hormonal alterations caused by the consumption of aripiprazole, possibly because this drug develops fewer hormonal effects than other AAPs. There are multicentric studies that evaluate the tolerability, efficacy, and safety of aripiprazole in schizophrenia and other mood disorders for up to 52 weeks of treatment (15 mg/day) (279–282). The administration of aripiprazole (15 mg/day) is recommended for the control of HPRL associated to chronic consumption of other AAPs such as risperidone, amisulpride, olanzapine (270, 275, 283), and benzamide, and it helps to maintain improvement in the positive and negative symptoms of patients (134, 284, 285). In fact, aripiprazole is prescribed as a substitute for treatments with AAPs when the patients show no signs of clinical response or when they exhibit severe symptoms of sexual dysfunction associated with HPRL (134, 282, 286, 287). Although there are few cases of patients with an increase in PRL during treatment (288–290), aripiprazole is considered a safe drug.

There is minimal evidence on its metabolic activity, yet aripiprazole is known to play a partially protective role (291–295). Concerning research of aripiprazole-induced effects in animal models (Wistar rats) and cell lines (rHypoE-19), beneficial changes over metabolic parameters such as risk dyslipidemia and body weight have been found (72, 296).

Regarding the immune effects caused by aripiprazole consumption, there is evidence that shows this drug produces significant changes, such as cell count and changes in cytokine secretion, response to ROS, and gene expression. Although reports on the adverse effects of aripiprazole are scant compared to other AAPs, there is minimal evidence of its effect on the decrease in white blood cell count (297). A 10-year old with attention deficit hyperactivity disorder (ADHD) treated with aripiprazole (5 mg/day) showed a lower absolute neutrophil count (ANC). Additionally, a 50-year old Caucasian woman with schizophrenia developed neutropenia after aripiprazole consumption (15 mg/day) for 5 days (298), and a 21-year old Asian man with a conduct disorder showed a drop in WBC and neutropenia during aripiprazole treatment (297, 298). In all cases, the discontinuation of aripiprazole resulted in the normalization of WBC count and ANC, suggesting that the long-term bone marrow suppression by this drug plays a role in repeated antipsychotic consumption.

Some studies have shown that this drug affect cytokines secretion toward an anti-inflammatory profile: A meta-analysis involving 505 patients treated with aripiprazole showed a relationship between cytokine levels (TNF-α and IFN-γ) and their possible role as state and trait markers (86, 299). Another report described that aripiprazole consumption (5–30 mg/day, 3 months) reduced TNF-α, IL-8, IL-21, IL-13, IL-17, and fractalkine (CXCL1) levels in 31 first-episode psychotic patients; the effect in these molecules exhibited a positive correlation with clinical improvement (174). Another study also demonstrated a decrease in IL−1β, IL-6, TNF-α, IL-12, IL-23, IL-4, and IFN-γ under aripiprazole treatment with a dose from 10 mg/day (week 1) to a maximum of 30 mg/day (weeks 2, 3, and 4) (300).

*In vitro* studies confirm those data, since PBMC from healthy subjects and THP-1 cells incubated with aripiprazole (10–5 μM) exhibited a decrease in the expression of pro-inflammatory cytokines IL-1β, IL-6, and TNF-α and reduced the levels of IL-2, IL-9, IP-10 (CXCL10), and MIP-1β (CCL4) in the supernatant (217). The anti-inflammatory effect shown by this drug could be associated to the decrease in gene expression of cyclooxygenase (COX)-2 and inducible nitric oxide synthase (iNOS), causing lower levels of NO, prostaglandin 2 (PGE 2), and TNF-α (301). Furthermore, It is known that RAW264.7 cells treated with aripiprazole (20 μM) inhibited the interaction of the second messengers TAK1, MKK4, and MKK7 on AP-1, and, and Syk, which play a key role in the NF-κB signaling pathway (301). Aripiprazole also acts as an antioxidant improving the response to ROS. Studies in murine (2 mg/kg) and *in vitro* (5 μM) models showed that this drug increased the activity of glutathione peroxidase (GSH-Px) and superoxide dismutase (SOD) enzymes, promoting a decrease in the concentration of NO in supernatants and TNF-α, IL-1α, IL-2, and IL-10 in mice serum levels. This antioxidant activity is related to the input of intracellular [Ca^2+^], which allows for ROS regulation and the decrease in inflammation cytokines (302, 303).

Some evidence suggests that this drug also modifies the gene expression of relevant genes; an *in vitro* study using primary human adipose-derived stem cells (ADSCs) demonstrated that aripiprazole (100 ng/mL) increased the expression of key genes involved in cell cycle (*ANAPC2, CD14*), apoptosis (*BCL2)*, nuclear and transporter receptors (*PPAR*α*, PPAR*γ*, ABCA1, LEPR, INSR*), transcript factors (*CEBPA, SREBF1, NF-KB1*), signal transduction (*IRS1, SIRT1*), adipogenic markers, lipid metabolism, adipokines (*ADFP, FABPN, LPL, ACSL1, ADIPOQ, LEP*) and cytokines and chemokines (TNF-α, IL-1β, IL-8, MCP-1). These results support the role of AAPs in the recruitment of MQs to adipose tissue by increasing MCP-1 and the risk of metabolic syndrome associated with drug treatment (185). However, this drug showed no significant immunotoxic effects in ICR mice and C6 glioma and RAW264.7 cells (50 mg/kg) when no alterations in organs or cell lines were found (304).

In summary, there is little evidence on the hormonal and immune effects of aripiprazole, as well as its partially protective role (291–295). These effects allow aripiprazole to suitably treat schizophrenia and bipolar disorder (304) (see Table 2).

## Paliperidone

Paliperidone, or 9-hydroxy-risperidone (see Figure 1), is the most significant active metabolite of risperidone. The FDA approved this drug for the treatment of schizophrenia in 2006 (305). Paliperidone is a monotherapy drug for short-term and maintenance treatment of schizophrenia as well as monotherapy or adjunct drug for the short-term treatment of schizoaffective disorder (306–308). It has also been used in the treatment of bipolar disorder (309), borderline personality disorder (310), Huntington's disease (311), ASD, and ADHD (312); however, it has not been approved to treat any of these last clinical conditions. Paliperidone is a racemic mixture of (+)-paliperidone and (–)-paliperidone enantiomers that undergo minimal hepatic metabolism (44). The available pharmaceutical formulations of this drug are oral immediate-release formulation, oral extended-release (ER) formulation, and intramuscular depot formulation (305).

The therapeutic activity of paliperidone is comparable with that of risperidone itself; its action mechanism is unknown, but it likely acts through a combination of 5-HT_2A_ agonism and D2 receptor antagonism (see Table 1) (44). This drug is also active as an antagonist for other receptors such as D3, D4 dopaminergic receptors, 5-HT_1A_, 5-HT_1B_, and 5-HT_1D_ serotoninergic receptors, and α-1 and α-2 adrenergic receptors, although it also acts as agonist to 5-HT_2C_ and 5- H1 histaminergic receptors (45).

The immunoendocrine alterations cited in this section are related to paliperidone; effects on 9-hydroxy-risperidone by risperidone consumption and its subsequent metabolism are not mentioned. The most representative endocrine alteration reported after paliperidone consumption is the increase in PRL or HPRL (207, 308, 313, 314). This alteration can produce prolactin-related adverse effects (PRL-RAEs) or be asymptomatic (315).

HPRL induced by risperidone/paliperidone treatment in schizophrenic patients was presented in association with rs40184 and rs3863145 variants in the *SLC6A3* gene of blood leukocyte DNA (132). According to several reports, paliperidone produced high HPRL incidence when compared vs. other SGAs in adults (dosage 7.03 ± 3.63 mg/day) (111) and pediatric patients (130). Paliperidone consumption showed an association between PRL, sex, and age (113, 130), although Druyts and cols. reported no differences between females and males (314). This drug increased PRL levels, yet some reports have shown that the switch from risperidone or paliperidone ER to paliperidone palmitate treatment (PP, an intramuscular depot formulation) reduced PRL concentration (316, 317) as well as sexual dysfunction (316), a common PRL-RAE. Similarly, patients with sexual dysfunction presented higher PRL as compared with no sexual dysfunction patients (318). Adolescent patients with PRL-RAE showed higher PRL levels when compared against patients without PRL-RAE (1.5–12 mg/day) (319). According to the literature above, different formulations of paliperidone could cause this alteration in pediatric and adult patients. The precise mechanism by which paliperidone increases PRL levels is unclear; however, it corresponds to D2 receptor blockade (320).

There are a few reports that show changes in other hormonal profiles in patients during paliperidone consumption. Although other AAPs mentioned in this review induce dysregulation in glucose metabolism, paliperidone does not modify serum levels of insulin. The acute and chronic treatment with paliperidone did not alter serum insulin levels and β-cell function with the homeostatic model assessment (HOMA-B) (207, 313, 321). However, a case report showed increased insulin secretion, causing hypoglycemia in a schizophrenic female patient (322).

The reports on immune alterations induced by paliperidone consumption are a few yet diverse. Several cases of schizophrenic patients showed that paliperidone treatment decreased leukocyte counts. Monotherapy with paliperidone produced leukopenia and neutropenia (323); still, the combined use of paliperidone depot/risperidone (100–2 mg/day) resulted in leukopenia and lymphopenia but risperidone alone did not (159). Agranulocytosis was reported in a patient when switching from risperidone to paliperidone treatment (6 mg/day) (324). The treatment with paliperidone ER/valproic acid (12–1,000 mg/day) caused leukopenia and neutropenia in a patient with schizoaffective disorder (325). In all cases, the cytopenic alterations were normalized after discontinuing the consumption of paliperidone. Some proposed mechanisms of AP-induced blood dyscrasia, such as paliperidone, include direct bone marrow suppression, antibody formation against hematologic precursors, and peripheral WBC destruction (326).

Paliperidone increases BDNF concentration during acute treatment. The serum levels of BDNF in first-episode schizophrenia patients increased after a 12-week paliperidone treatment negatively correlated with a reduction rate of the positive and negative symptoms scale (PANSS) score (unspecified dose) (327). However, the paliperidone ER treatment during 8 weeks did not increase BDNF serum concentration (unspecified dose) (328).

In blood, peripheral cells of patients with EPS (acute dystonia and drug-induced parkinsonism) showed a constructed network enriched in different biological processes related to pathways of NF-κB, an important transcription factor for immune response, (12.85 ± 2.85 mg/day) (329) in patients with paliperidone or risperidone treatment. *In vitro*, U-937 human cell line decreased cell survival with 25 and 50 μM/mL of paliperidone (330) (see Table 2).

## Asenapine

The FDA approved asenapine for the treatment of schizophrenia (331) and bipolar disorders (332) in 2019. This drug is a new AAP with unique features that was introduced in Japan in 2016, and it is the only AP used sublingually; its chemical structure of (±)-Asenapine can be described as a tetracyclic framework wherein N-methylpyrrolidine ring fuses at third and fourth positions with chlorophenyl phenyl ether in a trans geometry (333) (see Figure 1). This drug is metabolized rapidly in a process mediated by glucuronidation and demethylation pathways that induce two non-active metabolites, asenapine N-glucuronide and asenapine N-desmethyl carbamoyl glucuronide (334). Asenapine has subnanomolar and nanomolar affinities for diverse and numerous subtypes of aminergic G protein coupled receptors (GPCRs) associated to 5-HT, norepinephrine (NE), DA, and histamine (H) (335, 336). Still, the antagonist activity at 5-HT_1A_, 5-HT_1B_, 5-HT_2A_, 5-HT_2C_, 5-HT_5A_, 5-HT_6_, and 5-HT_7_ may contribute to the antimanic and antidepressant effects of asenapine (see Table 1) (39).

Endocrine deleterious side effects induced by asenapine consumption were reported in PRL and insulin blood levels. Asenapine displays more potent antagonist activity toward 5-HT_2A_ receptor than D2 receptor (337, 338), that is why it has a low propensity to cause PRL elevation (331, 339, 340); Therefore, this drug is one of the AP treatments of choice for breast cancer patients (341). Nevertheless, research groups reported that 2.3% of patients with bipolar disorder who received asenapine monotherapy had PRL levels ≥ 4 times the upper limit of the normal range, compared with those who received a placebo (332, 342), In contrast, 9% of patients with schizophrenia who received asenapine (5 and 10 mg twice daily) had PRL levels over 2-fold the upper limit of the normal range compared with those who received a placebo (343).

Insulin altered levels are associated with glucose metabolism disturbances and the evidence shows that asenapine modifies the blood levels of these hormones. In 302 patients (aged 10–17 years) with bipolar I disorder in manic or mixed episodes who were treated with asenapine (2, 5, or 10 mg twice, daily) for 3 weeks, the mean change from baseline in fasting insulin was significant when compared to controls. In all cases, the patients treated with asenapine increased their body weight (344). Contrastingly, no changes in insulin resistance were detected in adult female Sprague Dawley rats treated with asenapine (0.01, 0.05, 0.1, 0.5, 1.0 mg/kg) (67).

As described above, asenapine can interact with 5-HT, NE, DA, and H receptors expressed in leukocytes (28, 29, 345, 346). Then, the administration or consumption of this drug could induce changes in the inflammatory response in patients, although the evidence of this effect is very scarce. There is only one report of a case of pityriasis rosea secondary to asenapine consumption. A biopsy of the lesions evidenced superficial and deep perivascular and interstitial dermatitis with eosinophils and dermal perivascular lymphocytic infiltrate, as well as minimal parakeratosis and spongiosis (347). Although there was no molecular explanation of this phenomenon, we may speculate that this patient had an alteration in neurotransmitter receptors (density or functional alteration) expressed by leukocytes, becoming more susceptible to this aberrant inflammatory response secondary to asenapine consumption (see Table 2).

## Microbiota

Little is known about the effects of AAPs on the microbiota; however, a small body of evidence suggests they cause severe adverse effects. Olanzapine and risperidone induced an increase in Firmicutes and a decrease in *Bacteroidetes*, as well as metabolic alterations as a result of a shift toward a potentially obesogenic bacterial profile associated with short-chain fatty acids and inflammation in adults (348), children (349, 350), and rodents (351). These changes were also gender-dependent (349, 352, 353), with females showing a higher pro-inflammatory cytokine (IL-8 and IL-1β) response in circulation and macrophage infiltration; still, microbiota dysbiosis was equally present in males and females.

AAPs have a potent antibiotic effect, inducing a profound dysbiosis in the gut microbiota, either chronically or after short-term administration (205). Antibiotic co-administration resulted in further changes in microbiota composition. Interestingly, these antibiotic-dependent changes in microbiota diversity reduced the side effects, including macrophage infiltration. Furthermore, experiments in germ-free mice showed no alteration in their metabolic profile (352–355), indicating a clear role of the microbiota in the metabolic dysfunction associated with AAPs (352). Finally, fecal transplants from risperidone-treated mice induced excess weight gain in control mice (354). These alterations have been associated to a decrease in *Bifidobacterium, Escherichia coli*, and *Lactobacillus* and an increase in *Clostridium coccoides* (353).

Risperidone *in vitro* altered the colon microbiota just 24 h after administration, inducing specific metabolites (350). Probiotic treatment has shown a protective effect, restoring the *Bacteroidetes:Firmicutes* ratio, without reducing the AAPs effect (356).

## Epilog

The bidirectional communication between the SNC with other peripheral systems occurs by the release of soluble molecules that interact with their receptors. Any cell in the organism that bears a functional receptor for a molecule will respond when they interact. The complex structure that confers pharmacological non-specificity to AAPs allows for the interaction with the receptors they have an affinity for, not only in the CNS but also in all body cells. This result leads to the therapeutic effect of AAPs in various psychiatric conditions and their possible ability to modify the endocrine and immune systems as well as the gut microbiota. The therapeutic effect of AAPs is exhibited by the antagonism in CNS receptors that are involved in the pathophysiology of the disease. In schizophrenia, for example, the positive and negative symptoms decrease due to the AAP-receptor interaction in the mesocortical and mesolimbic pathways, although HPRL is caused by the antagonism of receptors in the tuberoinfundibular pathway. In addition, the antagonism of neurotransmitter receptors on leukocytes and glandular cells have immune and endocrine effects. The effect of each AAP is unique and depends on specificity and affinity characteristics.

AAPs are drugs prescribed for various psychiatric conditions due to their high efficiency and low rate of extrapyramidal effects. However, these drugs have systemic effects that are not only metabolic but also related to changes in endocrine and immune responses. Having greater knowledge of these immune, endocrine, and microbiota effects, allows clinicians to have a broader point of view and more significant criteria to prescribe these drugs to patients, considering that the adverse effects can modify the systemic response and generate undesirable effects, with a direct impact on the patients' quality of life. It is necessary to start a new generation of drugs that support the resolution of psychiatric symptoms with higher specificity to prevent acute adverse effects and the patients' systemic deterioration by chronic consumption.

## Author Contributions

SA-H, RE, and LP: conceptualization. All authors: writing-original draft preparation and writing-review and editing. LP: supervision.

## Conflict of Interest

The authors declare that the research was conducted in the absence of any commercial or financial relationships that could be construed as a potential conflict of interest.
