# Supplementary material for: Immunoendocrine Peripheral Effects Induced by Atypical Antipsychotics
Source: Front Endocrinol (Lausanne). 2020 Apr 21;11:195. doi: 10.3389/fendo.2020.00195 (PMC7186385; doi:10.3389/fendo.2020.00195)
Supplement: Supplementary file 1 [file Data_Sheet_1.docx]

**Supplementary Material**

**Iloperidone**

Iloperidone (piperidinyl-benzisoxazole) is a molecule derived from risperidone (1,2) (see figure 1). In 2009, the FDA approved this AAP for the treatment of the acute phase of schizophrenia in adults (3,4) and it is recommended until the stabilization phase (5). The therapeutic effect of this drug is associated with the agonism of the 5-HT_1A_, 5-HT_2A_, 5-HT_2C_, 5-HT_6_, 5-HT_7_, D1-4, α-2A, and H1 receptors (see Table 1) (6–11). It is metabolized primarily by carbonyl reduction, hydroxylation mediated by CYP2D6, and O-demethylation (mediated by CYP3A4). There are two predominant metabolites, P95 and the active metabolite P88.

As other AAPs, iloperidone is able to alter the serum levels of PRL (12) due to its affinity for DA receptors (6) and it seems the effect of this drug on PRL depends on the consumption time and the dose, yet there is evidence that shows no changes in PRL levels during iloperidone consumption (13). In contrast, there is a case report of HPRL with galactorrhea in a middle-aged woman treated with iloperidone (8 mg/day) for three months (14). A study showed decreased PRL in serum (24 mg/day) among 86 patients with schizophrenia after 25 weeks of treatment (15). Three prospective trials (n = 1,943) with three different iloperidone dose ranges (4–8, 10–14, and 20–24 mg/day) for six weeks showed that low and medium doses (4–8 and 10–14 mg/day) decreased serum PRL in patients. However, PRL levels were not detected in patients with high dose (20–24 mg/day) compared with baseline (16). This effect is associated to the interaction between iloperidone and D2 receptors on the tuberoinfundibular pathway, so the DA cannot inhibit PRL secretion in the anterior hypophysis. These changes are associated with genetic factors and drug dose, which explains the results of the studies mentioned above (14,16) (see figure 2).

There are no more reports that exhibit the presence of hormonal alterations induced by iloperidone consumption and there is no evidence showing alterations in the humoral or cellular inflammatory response during iloperidone consumption. Further studies are required to evaluate the effect of this drug on the endocrine function and the impact of its consumption on the inflammatory response and explain the molecular mechanisms involved in the possible alterations.

**Lurasidone**

Lurasidone, a benzothiazole derivative (see figure 1), is used as an AP treatment in adults. This drug gained the FDA approval for the treatment of schizophrenia in 2010 (17) and bipolar depression both in monotherapy and as adjunctive therapy with lithium or valproate in 2013 (18). Lurasidone is metabolized through the CYP3A4 pathway and its consumption with food is recommended for greater efficiency (19). Its metabolism pathways are oxidative N-dealkylation, hydroxylation of norbornane ring, and S-oxidation. This drug is broken down into three active and two inactive metabolites (17).

Lurasidone has a greater affinity for 5-HT_7_, D2, 5-HT_2A_, 5-HT_1A_, and adrenergic α2c receptors. It also has moderate affinity for adrenergic receptors α1 and α2A, weak affinity for D1 and 5-HT_2C_, and negligible affinity for histamine H1, muscarinic, nicotinic, glutamate, and sigma receptors, as well as dopamine and serotonin transporters (See Table 1) (20).

Although it is reported to induce modest, dose-dependent PRL and HPRL elevations, especially at the beginning of treatment, as well as HPRL in some patients, lurasidone seems to be associated with no clinically meaningful PRL alterations in most cases (21). PRL alterations are associated with the fast dissociation from the D2R and the PRL-sparing properties of APs. Lurasidone is associated with lesser degrees of PRL elevation since it shows a fast D2R dissociation (21). There are a few reports about the effect of this drug on the endocrine system; however, the benign metabolic profile during treatment and its minimal effects on body weight, glucose, and lipid concentrations provide lurasidone with an advantage over other SGAs (22).

Some reports prove the effect of lurasidone consumption in the inflammatory response. One of these alterations is the cell count. Although lurasidone is less likely to cause side effects, a 29-year old patient with bipolar depression developed thrombocytopenia after a 3-month treatment (80 mg/day) (23). A second report linked neutropenia in patients in remission from a manic episode with the use of 40 mg/day of lurasidone (24).

Lurasidone also affects soluble mediators of inflammatory response. It has been proven that patients with bipolar depression treated with 20–60 mg/day (n=161) and 80–120 mg/day (n=162) lurasidone showed a significantly decreased C-reactive protein (CRP) after a 6-week treatment. Although this is not directly related to the effect of the drug upon inflammatory pathways, it is generally accepted that lurasidone plays a regulatory role in central DA levels, promoting depression improvement (25) (see figure 2). In light of the importance of Lurasidone in the immune and endocrine system, additional studies will be needed to take advantage of the clinical potential of this drug.

**Bibliography**

1. Strupczewski JT, Bordeau KJ, Chiang Y, Glamkowski EJ, Conway PG, Corbett R, Hartman HB, Szewczak MR, Wilmot CA, Helsley GC. 3-[[(Aryloxy)alkyl]piperidinyl]-1,2-Benzisoxazoles as D2/5-HT2 Antagonists with Potential Atypical Antipsychotic Activity: Antipsychotic Profile of Iloperidone (HP 873). *J Med Chem* (1995) **38**:1119–1131. doi:10.1021/jm00007a009

2. Marino J, Caballero J. Iloperidone for the Treatment of Schizophrenia. *Ann Pharmacother* (2010) **44**:863–870. doi:10.1345/aph.1M603

3. Crabtree BL, Montgomery J. Iloperidone for the Management of Adults with Schizophrenia. *Clin Ther* (2011) **33**:330–345. doi:10.1016/j.clinthera.2011.03.006

4. Arif SA, Mitchell MM. Iloperidone: A new drug for the treatment of schizophrenia. *Am J Heal Pharm* (2011) **68**:301–308. doi:10.2146/ajhp100079

5. Scott L. Iloperidone: In Schizophrenia. *CNS Drugs* (2009) **23**:867–880.

6. Kalkman HO, Subramanian N, Hoyer D. Extended radioligand binding profile of iloperidone: A broad spectrum dopamine/serotonin/norepinephrine receptor antagonist for the management of psychotic disorders. *Neuropsychopharmacology* (2001) **25**:904–914. doi:10.1016/S0893-133X(01)00285-8

7. Kalkman HO, Feuerbach D, Lötscher E, Schoeffter P. Functional characterization of the novel antipsychotic iloperidone at human D2, D3, α2C, 5-HT6, and 5-HT1A receptors. *Life Sci* (2003) doi:10.1016/S0024-3205(03)00419-3

8. Richelson E, Souder T. Binding of antipsychotic drugs to human brain receptors focus on newer generation compounds. *Life Sci* (2000) **68**:29–39. doi:10.1016/s0024-3205(00)00911-5

9. Roth BL, Lopez E, Patel S, Kroeze WK. The multiplicity of serotonin receptors: Uselessly diverse molecules or an embarrassment of riches? *Neuroscientist* (2000) **6**:252–262. doi:10.1177/107385840000600408

10. Kongsamut S, Roehr JE, Cai J, Hartman HB, Weissensee P, Kerman LL, Tang L, Sandrasagra A. Iloperidone binding to human and rat dopamine and 5-HT receptors. *Eur J Pharmacol* (1996) **317**:417–423. doi:10.1016/S0014-2999(96)00840-0

11. Caccia S, Pasina L, Nobili A. New atypical antipsychotics for schizophrenia: iloperidone. *Drug Des Devel Ther* (2010) **4**:33–48. Available at: http://www.embase.com/search/results?subaction=viewrecord&from=export&id=L358687725%0Ahttp://www.dovepress.com/getfile.php?fileID=5766

12. Citrome L. Iloperidone redux: A dissection of the drug approval package for this newly commercialised second-generation antipsychotic. *Int J Clin Pract* (2010) **64**:707–718. doi:10.1111/j.1742-1241.2010.02344.x

13. Cutler AJ, Kalali AH, Weiden PJ, Hamilton J, Wolfgang CD. Four-week, double-blind, placebo- and ziprasidone-controlled trial of iloperidone in patients with acute exacerbations of schizophrenia. *J Clin Psychopharmacol* (2008) **28**:20–28. doi:10.1097/JCP.0b013e318169d4ce

14. Dutta A, Barua S, Dan A, Chakraborty K, Mandal M. Iloperidone-induced galactorrhea in a middle-aged female. *Indian J Psychol Med* (2012) **34**:396–398. doi:10.4103/0253-7176.108233

15. Cutler AJ, Kalali AH, Mattingly GW, Kunovac J, Meng X. Long-term safety and tolerability of iloperidone: results from a 25-week, open-label extension trial. *CNS Spectr* (2013) **18**:43–54. doi:10.1017/S1092852912000764

16. Weiden PJ, Cutler AJ, Polymeropoulos MH, Wolfgang CD. Safety profile of iloperidone: A pooled analysis of 6-week acute-phase pivotal trials. *J Clin Psychopharmacol* (2008) **28**: doi:10.1097/JCP.0b013e3181694f5a

17. Jaeschke RR, Sowa-Kućma M, Pańczyszyn-Trzewik P, Misztak P, Styczeń K, Datka W. Lurasidone: The 2016 update on the pharmacology, efficacy and safety profile. *Pharmacol Rep* (2016) **68**:748–55. doi:10.1016/j.pharep.2016.04.002

18. Bawa R, Scarff JR. Lurasidone: A new treatment option for bipolar depression— A review. *Innov Clin Neurosci* (2015) **12**:21–23. Available at: http://www.ncbi.nlm.nih.gov/pubmed/25852975 [Accessed March 3, 2020]

19. Tarazi FI, Riva MA. The preclinical profile of lurasidone: Clinical relevance for the treatment of schizophrenia. *Expert Opin Drug Discov* (2013) doi:10.1517/17460441.2013.815163

20. Ishibashi T, Horisawa T, Tokuda K, Ishiyama T, Ogasa M, Tagashira R, Matsumoto K, Nishikawa H, Ueda Y, Toma S, et al. Pharmacological profile of lurasidone, a novel antipsychotic agent with potent 5-hydroxytryptamine 7 (5-HT 7 ) and 5-HT 1A receptor activity. *J Pharmacol Exp Ther* (2010) doi:10.1124/jpet.110.167346

21. Peuskens J, Pani L, Detraux J, De Hert M. The effects of novel and newly approved antipsychotics on serum prolactin levels: A comprehensive review. *CNS Drugs* (2014) doi:10.1007/s40263-014-0157-3

22. Krause M, Zhu Y, Huhn M, Schneider-Thoma J, Bighelli I, Chaimani A, Leucht S. Efficacy, acceptability, and tolerability of antipsychotics in children and adolescents with schizophrenia: A network meta-analysis. *Eur Neuropsychopharmacol* (2018) doi:10.1016/j.euroneuro.2018.03.008

23. Rafi M, Goyal C, Reddy P, Reddy S. Lurasidone induced thrombocytopenia: Is it a signal of drug induced myelosuppression? *Indian J Psychol Med* (2018) doi:10.4103/IJPSYM.IJPSYM_374_17

24. Singh S, Ahmad H, John AP. Lurasidone associated neutropenia. *Aust N Z J Psychiatry* (2017) doi:10.1177/0004867417708869

25. Raison CL, Pikalov A, Siu C, Tsai J, Koblan K, Loebel A. C-reactive protein and response to lurasidone in patients with bipolar depression. *Brain Behav Immun* (2018) doi:10.1016/j.bbi.2018.08.009
